# Electroretinographic evidence suggesting that the type 2 diabetic retinopathy of the sand rat *Psammomys obesus* is comparable to that of humans

**DOI:** 10.1371/journal.pone.0192400

**Published:** 2018-02-08

**Authors:** Ahmed Dellaa, Maha Benlarbi, Imane Hammoum, Nouha Gammoudi, Mohamed Dogui, Riadh Messaoud, Rached Azaiz, Ridha Charfeddine, Moncef Khairallah, Pierre Lachapelle, Rafika Ben Chaouacha-Chekir

**Affiliations:** 1 Laboratory of Physiopathology, Food and Biomolecules of the Higher Institute of Biotechnology Sidi Thabet, Manouba University, BiotechPole Sidi Thabet, Ariana, Tunisia; 2 Faculty of Sciences of Bizerte, Carthage University, Bizerte, Tunisia; 3 Department of functional explorations of the nervous system, University Hospital of Sahloul, Sousse, Tunisia; 4 Department of Ophthalmology, University Hospital of Fattouma Bourguiba, Monastir, Tunisia; 5 UNIMED Pharmaceutical Industry, industrial area Kalaa Kebira, Sousse, Tunisia; 6 Department of Ophthalmology, Research Institute of the McGill University Health Centre, Montreal, Quebec, Canada; Roskamp Institute, UNITED STATES

## Abstract

**Purpose:**

Type 2 diabetic retinopathy is the main cause of acquired blindness in adults. The aim of this work was to examine the retinal function of the sand rat *Psammomys obesus* as an animal model of diet-induced type 2 diabetes when subjected to a hypercaloric regimen.

**Materials and methods:**

Hyperglycemia was induced in *Psammomys obesus* by high caloric diet (4 kcal/g). The visual function of control (n = 7) and diabetic (n = 7) adult rodents were followed up during 28 consecutive weeks with full-field electroretinogram(ERG) recordings evoked to flashes of white light according to the standard protocol of the International Society for Clinical Electrophysiology of Vision protocol (ISCEV).

**Results:**

Twenty-eight weeks following the induction of diabetes, results revealed significantly reduced and delayed photopic and scotopic ERG responses in diabetic rats compared to control rats. More specifically, we noted a significant decrease in the amplitude of the dark-adapted 0.01ERG (62%), a- and b-wave amplitudes of the dark-adapted 3.0 ERG (33.6%, 55.1%) and the four major oscillatory potentials components (OP1-OP4) (39.0%, 75.2%, 54.8% and 53.7% respectively). In photopic conditions, diabetic rats showed a significant decrease in a- and b-wave (30.4%, 43.4%), photopic negative response (55.3%), 30 Hz flicker (63.7%), OP1-OP4(51.6%, 61.8%, 68.3% and 47.5% respectively) and S-cone (34.7%). Significantly delayed implicit times were observed for all ERG components in the diabetic animals. Results obtained are comparable to those characterizing the retinal function of patients affected with advanced stage of diabetic retinopathy.

**Conclusion:**

*Psammomys obesus* is a useful translational model to study the pathophysiology of diabetic retinopathy in order to explore new therapeutic avenues in human patients.

## Introduction

Diabetic retinopathy (DR), which can lead to blindness in severe cases, is reported to affect more than 90% of diabetic patients [1]. The pathophysiology of diabetic retinopathy is believed to result from the sustained exposure to hyperglycemia which leads to retinal biochemical abnormalities [2]. In addition, although DR has long been recognized as a vascular disease [3–5], the neuronal cells of the retina are also affected [6–10]. Supportive of the latter, previous studies reported that some visual anomalies such as color vision deficits [11, 12] or decreased contrast sensitivity [13] precede the vascular signs of DR, suggesting that the vascular abnormalities may not be the first sign characterizing the onset of DR [14, 15]. Similarly, it was previously shown that flash and multifocal electroretinograms can detect retinal neurosensory changes long before an observable retinopathy occurs [16–18]. For instance, it was shown that the onset of the proliferative phase of diabetic retinopathy was better predicted with the selective amplitude reduction of the oscillatory potentials of the ERG than with the vascular lesions seen in fundus photographs [19, 20]. Clinical studies have also shown that retinal ganglion cells (RGC) and neuronal activity progressively decreases with advancing DR as indicated by the photopic negative response (PhNR) that follows the photopic ERG b-wave [14]. Chemically-induced diabetes was also shown to yield early functional changes in the retina, such as in streptozotocin (STZ)-treated rodents [21–24] and alloxan-treated rabbits [25, 26].

We have previously shown that sand rats *Psammomys obesus* (*P*.*obesus*) fed with a high caloric diet will spontaneously develop type 2 diabetes, with accompanying retinopathy, thus making them a valid translational model of this retinal disorder [27]. Animals subjected to this regimen typically develop hyperglycemia and severe DR-like ocular complications such as vascular alterations, elevated vitreous ratios of pro- and anti-angiogenic growth factors, blood-retinal barriers structure breakdown and neural and glial changes similar to the ones occurring in humans [27, 28]. The purpose of this study was to investigate if the diabetes-induced retinal functional changes observed in *P*.*obesus* also compare with what is reported in diabetic human subjects.

## Materials and methods

### Animals

*P*.*obesus* was captured in a semi-desertic area of southern Tunisia in Gafsa (National Park of Bou-Hedma), in strict accordance with the national regulations on the treatment of wildlife. All *P*.*obesus* were housed in standard animal housing rooms on 12/12-hour light/dark cycle with food and water *adlibitum*. The room temperature and relative humidity was set simultaneously at 24°C ± 1°C and 70% ± 5%. Only male rats were used in this study. Data for this study came from 14 adult males separated into two groups. The control group (n = 7) received a natural hypocaloric (0.4 kcal/g wet weight) vegetable diet, i.e., halophilic plants (Chenopodiaceae), rich in water and mineral salts. The diabetic group (n = 7) received a standard laboratory rat enriched chow feed (4 kcal/g) and mineral water *ad libitum*. The animals were followed up for a maximum of 7 months with measurements of body weight and plasmatic glucose. All procedures adhered to the ARVO Statement for the Use of Animals in Ophthalmic and Vision Research and approved by the local Bio-Medical Ethic Committee of Pasteur Institute of Tunis (protocol # 2016/11/E/ISBST/V1). *P*.*obesus* was captured according to the rules authorization of Tunisian Agriculture Ministry (ref. 2016/1693).

### Electroretinography

Measurements were made as described in our previous study [29]. Briefly, the visual monitor system (Mon Color, Metrovision) was used for stimulus generation and data acquisition. *P*.*obesus* were dark-adapted overnight, and the experimental procedure was done under a dim-red light (<1 Lux). Animals were anesthetized with an intraperitoneal injection of ketamine (120 mg/kg). The pupils were dilated using tropicamide (25mg/5ml; UNIMED, Tunisia) and the cornea was anesthetized with a drop of 0.5% Alcaine. For all animals, the ERG was recorded with a DTL fiber electrode (X-static silver coated conductive nylon yarn, Sauquoit Industries, Scranton, PA, USA) maintained on the cornea with one drop of tear gel (Lacryvisc, Carbomer 974 P, Alcon), to prevent corneal dryness. The reference and ground stainless steel needle electrodes were inserted subcutaneously on the forehead and tail, respectively. The five standard ERG responses advocated by the International Society for Clinical Electrophysiology of Vision (ISCEV) were used to compare the retinal function of the control and diabetic *P*.*obesus* as well as human subjects [30].

The scotopic ERG responses (Amplification: x 12500; 1–1200 Hz bandwidth) were evoked to flashes of white light of 0.01cd.s/m^2^ (rod responses) and 3cd.s/m^2^ (mix rod-cone response and oscillatory potentials: OPs). Oscillatory potentials were obtained by Fourier transform using an 80–200 Hz bandwidth which is a software filter built in the instrument. Following the scotopic recordings and after 10 minutes of light-adaptation (30 cd.m-^2^ background light), photopic (cone-mediated) ERGs were evoked using flashes of white light of 3 3cd.s/m^2^ in intensity. Each set of ERG responses was an average of 20 responses evoked at an inter-stimulus interval of 1 second[29]. A 30-Hz flicker response was also obtained using the same flash intensity and background. The S-cone response was obtained to a violet flash (414 nm) stimulus with intensity of 0.0045cd s/m2 delivered against a red-orange (595 nm) background 150 cd.m-^2^ to suppress the contribution of rods and M cones.

### Data analysis

ERG analysis was performed according to the standard practice [30] as previously reported [29]. The following ERG parameters were measured: rod-response amplitude (from the baseline to positive peak), a-wave amplitude (baseline to the first negative deflection), b-wave amplitude (a-wave peak to positive b-wave peak), i-wave amplitude (from the trough of the b-wave to the peak of the i-wave), PhNR amplitude (baseline to negative trough following b-wave), 30 Hz flicker amplitude (from the trough to the peak), OPs amplitude (the sum amplitude of wavelets 1–4 measured from the trough to the peak of each response component), S-cone amplitude (the b-wave amplitude of the S-cone response was quantified) and the b/a-wave amplitude ratio. All peak times were measured from the flash onset to the peak of each component.

Data was expressed as means ± standard error of mean (SEM). Comparison findings between groups were done using the Mann-Whitney test. Differences were considered statistically significant when the *p*-values were less than 0.05. All statistical analyses were performed with GraphPad Prism (GraphPad Software Inc., San Diego, USA).

## Results

### Diabetes affects biological parameters

**Fig 1:**
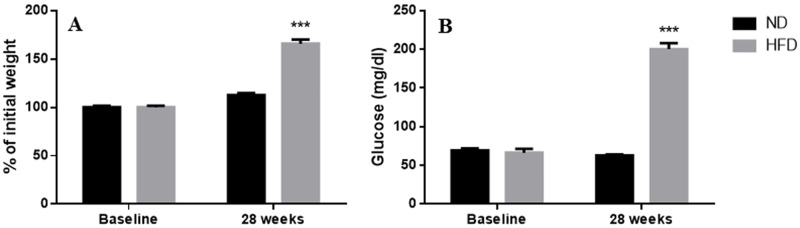
Body weight in % of initial weight (A) and blood glucose (B) parameters before and after normal diet (ND; N = 7 *P*.*ob*) and high fat diet (HFD; N = 7 *P*.*ob*) feeding during 28 weeks in *Psammomys obesus*. Values are given as means ± SEM. *** *p* ≤ 0.001. Control (black trace); Diabetic (grey trace).

At Fig 1 the average body weight in % (A) and blood glucose parameters levels (B)obtained measured 28 weeks following the induction of diabetes are presented. At the baseline, the body weight and blood glucose levels were similar between the two groups. Twenty-eight weeks of high-fat diet increased body weight by 65.9% compared to the baseline and by 12.6% compared with control-fed on with the natural diet. Similarly, the blood glucose level was significantly increased (200.3 ± 7.7 vs 62.7 ± 1.6 mg/dl; *p* = 0.0006) in the high-fat diet group compared to the natural diet group.

### The effect of diabetes progression on the ERG

**Fig 2:**
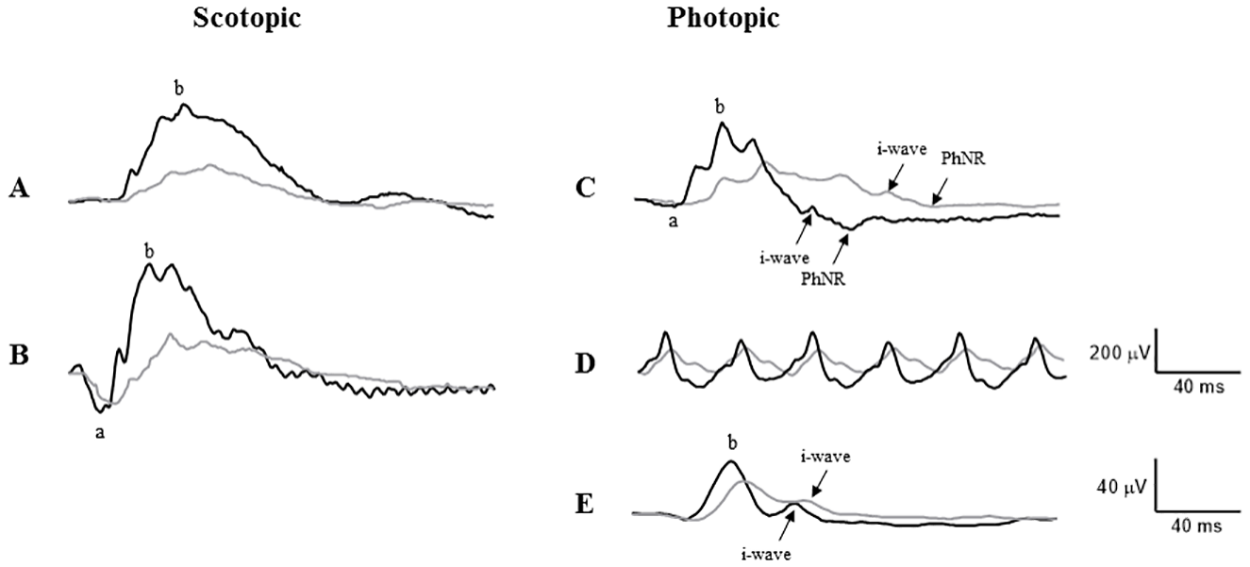
Retina function (electroretinograms) of *Psammomys obesus*, control and diabetic, at 28 weeks following the onset of diabetes. Representative traces indicating: (A) Rod responses using 0.01 cd.s/m^2^ flash. (B) mixed response using 3 cd.s/m^2^ flash. (C) Photopic responses using 3 cd.s/m^2^ flash. (D) Photopic 30 Hz flicker response. (E) Photopic S-cone response using 0.0045cd.s/m^2^ blue flash on an orange background. Individual waveform components are indicated in parentheses. Black traces—responses from control animals, Grey traces—from diabetic. For more details, see main text.

As illustrated in Fig 2 (and summarized in Table 1), our results show that following 28 weeks of diabetes all the ERG components of the photopic and scotopic responses (including the scotopic but not the photopic b/a wave ratios) were significantly attenuated (S7–S12 Figs) and delayed compared to control (S1–S6 Figs).

**Table 1:** ERG components for control and diabetic *Psammomys obesus*.

ERG Parameters	Control (n = 7)	Diabetic (n = 7)	*p*-values
Rod response			
Amplitude^α^	398.8 ± 22.2	146.1 ± 4.1	*** 0.0006
Implicit time^β^	53.1 ± 1.3	61.4 ± 2.4	** 0.01
Scotopic b-wave			
Amplitude	647.1 ± 25.4	290.7 ± 11.5	*** 0.0006
Implicit time	35.0 ± 1.9	49.6 ± 2.7	*** 0.007
Scotopic a-wave			
Amplitude	163.1 ± 6.5	108.5 ± 9.8	*** 0.0006
Implicit time	14.0 ± 0.5	21.0 ± 0.4	*** 0.0006
Scotopic b-/a- wave amplitude ratio	4.0 ± 0.2	2.8 ± 0.2	*** 0.002
Dark-adapted OP			
OP1 amplitude	29.7 ± 3.4	16.9 ± 0.6	** 0.01
OP2 amplitude	136.6 ± 7.0	32.4 ± 9.4	*** 0.0006
OP3 amplitude	117.3 ± 17.2	43.7 ± 6.8	*** 0.002
OP4 amplitude	59.1 ± 9.0	25.8 ± 4.0	*** 0.002
Sum OP amplitude	342.8 ± 30.5	117.0 ± 18.2	*** 0.0006
OP1 implicit time	14.2 ± 0.1	16.3 ± 0.6	** 0.01
OP2 implicit time	22.9 ± 0.5	28.1 ± 0.7	*** 0.006
OP3 implicit time	32.3 ± 0.8	38.3 ± 1.3	*** 0.002
OP4 implicit time	43.1 ± 0.8	49.4 ± 4.6	** 0.01
Photopic a-wave			
Amplitude	28.1 ± 1.1	19.4 ± 0.6	*** 0.001
Implicit time	15.9 ± 0.3	20.4 ± 0.7	**** 0.0001
Photopic b-wave			
Amplitude	308.3 ± 22.4	169.7 ± 24.6	*** 0.007
Implicit time	39.1 ± 0.7	60.2 ± 6.3	*** 0.0006
Photopic b-/a- wave amplitude ratio	11.1 ± 1.1	8.7 ± 1.1	0.1
i-wave			
Amplitude	27.1 ± 3.1	12.4 ± 0.7	* 0.04
Implicit time	78.5 ± 1.5	107.8 ± 8.8	*** 0.008
PhNR			
Amplitude	88.4 ± 7.4	37.8 ± 4.4	*** 0.002
Implicit time	97.5 ± 1.8	133.9 ± 7.7	*** 0.004
Photopic adapted OP			
OP1 amplitude	24.1 ± 1.8	11.2 ± 0.6	*** 0.001
OP2 amplitude	48.1 ± 6.7	17.4 ± 1.0	*** 0.0006
OP3 amplitude	54.9 ± 4.3	16.6 ± 2.5	*** 0.0006
OP4 amplitude	43.8 ± 10.1	18.1 ± 3.6	** 0.01
Sum OP amplitude	170.9 ± 17.7	63.3 ± 6.1	*** 0.0006
OP1 implicit time	16.4 ± 0.8	25.2 ± 1.0	*** 0.0006
OP2 implicit time	27.4 ± 0.8	35.3 ± 0.5	*** 0.001
OP3 implicit time	37.4 ± 0.7	45.4 ± 2.1	*** 0.001
OP4 implicit time	48.3 ± 1.2	56.6 ± 3.8	** 0.06
30-Hz flicker			
Amplitude	293.6 ± 16.4	105.7 ± 7.3	*** 0.0006
Implicit time	34.1 ± 0.2	36.5 ± 0.4	** 0.01
Photopic S-cone			
Amplitude	41.8 ± 5.2	27.1 ± 3.1	*** 0.001
Implicit time	43.4 ± 0.7	50.5 ± 2.3	** 0.01

^α^Amplitude is in microvolts (μV)

^β^Implicit time is in milliseconds (ms)

Data are expressed as the mean ± SEM.

* *p* ≤ 0.05

** *p* ≤ 0.01

*** *p* ≤ 0.001

**** *p* ≤ 0.0001

**Fig 3:**
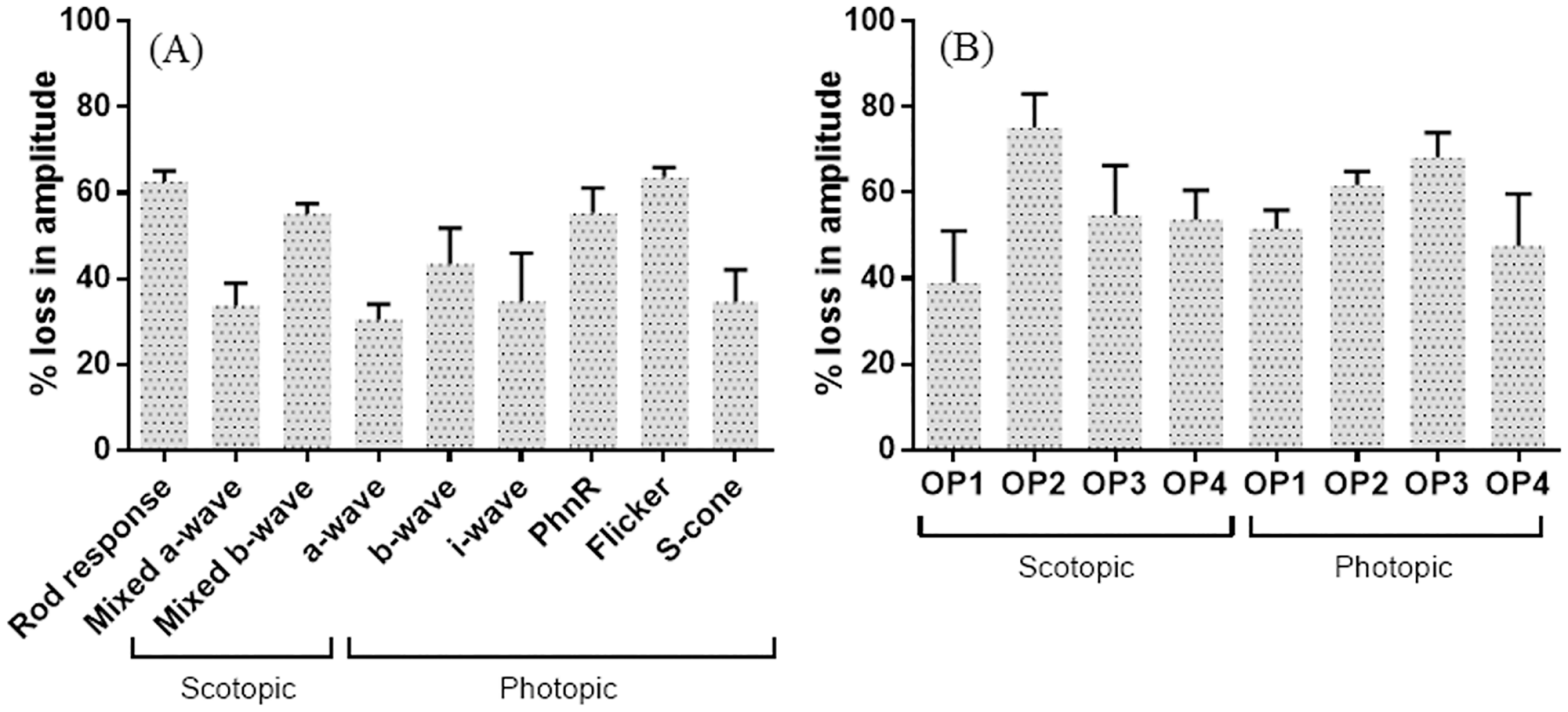
Percentage loss of amplitude in various ERG waveforms in diabetic *P*.*obesus* after 28 weeks. Left panel (A) indicates changes in main wave form parameters; right panel (B) indicates changes in individual oscillatory wavelets. Each bar graph indicates average ± SEM.

Fig 3 displays the percentage of diabetes-induced amplitude decrease in different ERG components. Compared to the control group, the amplitude of the rod-response and the mixed a-wave and b-wave (rod-cone) decreased significantly by 62.6% (p = 0.0006), 33.6%(p = 0.0006) and 55.1% (p = 0.0006), respectively (Table 2). Likewise, in photopic conditions the amplitude of a-, b-, i-wave, PhNR, flicker and S-cone were also significantly decreased between 30.4% and 68% (Table 2). There were no significant differences in the amount of diabetes-induced amplitude reductions when photopic and scotopic ERG components were compared (Table 3).

**Table 2:** The amplitudes percentage decrease in various ERG waveforms in diabetic *P*.*obesus* compared to control group.

ERG waveforms	Percentage loss in amplitude (%)
Scotopic condition	
Rod response	62.6 ± 2.5
Mixed a-wave	33.6 ± 5.3
Mixed b-wave	55.1 ± 2.4
OP1	39.0 ± 12.1
OP2	75.2 ± 7.9
OP3	54.8 ± 11.6
OP4	53.7 ± 6.8
Sum OP	63.8 ± 7.1
Photopic condition	
a-wave	30.4 ± 3.6
b-wave	43.4 ± 8.4
i-wave	34.7 ± 11.2
PhNR	55.3 ± 5.9
Flicker	63.7 ± 2.2
S-cone	34.7 ± 7.4
OP1	51.6 ± 4.3
OP2	61.8 ± 3.1
OP3	68.3 ± 5.7
OP4	47.5 ± 12.2
Sum OP	60.9 ± 4.9

**Table 3:** Comparison between percentage change in amplitude of different ERG components.

Comparison	p-value	Comparison	p-value
Photopic vs scotopic responses		Other responses b-wave vs a-wave	
a-wave	0.52	Scotopic a-wave vs b-wave	0.07
b-wave	0.96	Photopic a-wave vs b-wave	0.18
OP1	0.66	Slow waves vs fast waves	
OP2	0.14	Scotopic a-wave vs sum OP	*0.01
OP3	0.31	Scotopic b-wave vs sum OP	0.05
OP4	0.66	Photopic a-wave vs sum OP	**0.001
		Photopic b-wave vs sum OP	0.16

* *p ≤* 0.05

** *p ≤* 0.01

N = 7 diabetic *P*.obesus.

**Fig 4:**
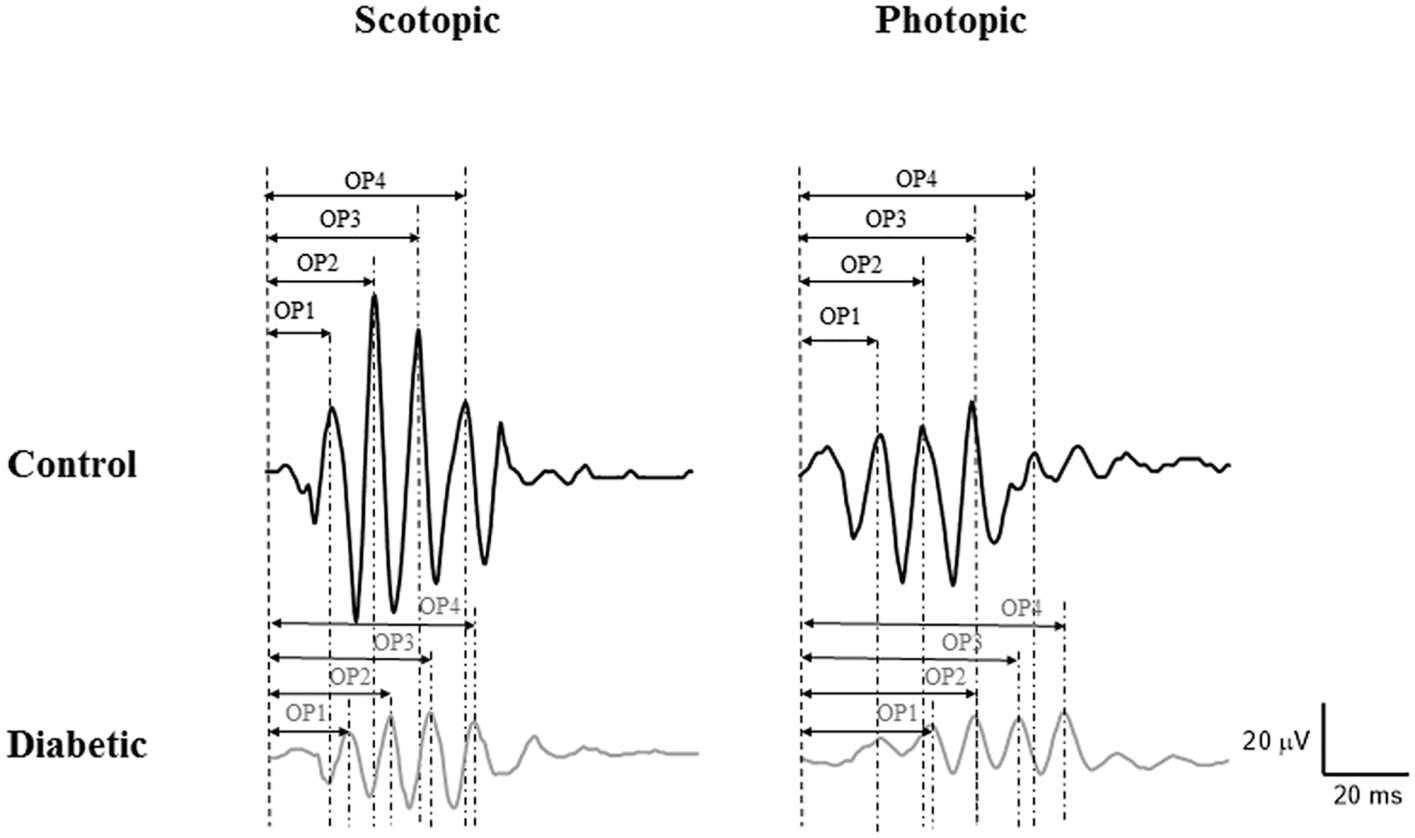
Individual representative of oscillatory potentials recorded from control and diabetic *P*.*obesus*. **Ops were recorded in response to 3 cd.s/m^2^ flash under scotopic and photopic conditions**. The OPs have been enumerated on each trace. Control (black trace); Diabetic (grey trace). Horizontal calibration, 20 ms; vertical calibration, 20 μV.

Results obtained with the OPs of control and diabetic *P*.*obesus* are presented at Fig 4. The summed amplitude of the scotopic OPs in diabetic *P*.*obesus* was approximately 62% smaller than control. When considered individually, the amplitudes of all OPs (OP1-OP4) were significantly (*p ≤* 0.05) reduced by 39.0%, 75.1%, 54.8% and 53.7% respectively. Similarly, the amplitudes of all photopic OPs (OP1- OP4) were significantly (*p ≤* 0.05) reduced by 51.6%, 61.8%, 68.3% and 47.5% respectively, as well as delayed implicit time of each oscillatory potential peak were noted (Fig 2; Table 1).

As shown in Table 3, there were no significant differences in the DR-induced amplitude reduction measured when the photopic and scotopic a-waves, b-waves and OPs were compared to each other. Similarly, when considering the scotopic response, both a-and b-waves were similarly attenuated and the same was also observed with the photopic response. However, of interest, while the DR-induced attenuation of the photopic and scotopic a-waves appeared to be significantly different from that of the corresponding OPs, photopic and scotopic b-waves and corresponding OPs were similarly attenuated. The latter would suggest that DR differentially affects the outer (a-wave) and inner (OPs more than b-wave) retina, regardless of the state of retinal adaptation.

## Discussion

*P*.*obesus* maintained on a high-fat diet for 28 consecutive weeks developed hyperglycemia with body weight increase as reported in a previous study of ours [31]. This high-fat diet induced a metabolic stress syndrome in *P*.*obesus* which led to an alteration to the retinal structure [31]. The present study demonstrated that this alteration to the retinal structure is paralleled by an equally profound deterioration in retinal function (decrease in amplitude scotopic and photopic a- and b-waves, OP1-OP4 components, PhNR, Flicker and S-cone).

Electrophysiological evaluation after the morphological assessment in diabetic *P*.*obesus* [27] was needed to clarify the functional changes in the photoreceptor cells of diabetic retina. Several studies used the *P*.*obesus* as an experimental model to study metabolic dysfunction leading to diabesity and its complications such as nephropathy, cardiomyopathy and retinopathy [27, 32, 33]. These metabolic and endocrine disorders are comparable to those observed in humans with type 2 diabetes. Our recent work [27] on the molecular and cellular alterations showed that the DR of *P*.*obesus* is similar to those reported in diabetic patients. Similarly, the electroretinogram of *P*.*obesus* also shares several features with that of human subjects [29], making it a remarkable model to investigate the retinal complications of diet-induced type 2 DR as it meets the criteria listed by the AMDCC [Animal models of diabetic complications consortium (www.amdcc.org)]. Of interest, in the present study, all *P*.*obesus* which developed DR after 28 weeks of high-fat treatment also developed a retinal dysfunction as measured with the ERG.

### Impairment of scotopic and photopic ERG parameters

Our results demonstrated that the amplitude of a- and b- waves ERG of diabetic rodents at 28weeks were significantly reduced, and the implicit times were considerably delayed compared to control *P*.*obesus*. These functional changes suggest that photoreceptor function, as well as synaptic transmission from the photoreceptors to bipolar cells, were affected *in vivo* by the high-fat induced hyperglycemia [27, 34]. The morphological findings after 7 months of diabetes in *P*.*obesus* showed a decreased expression of both PKC α and ζ isoforms that are Ca2+ independent and co-localized in rod bipolar cells[27]. This decreased expression may be related to photoreceptor alterations in diabetic *P*.obesus retina. Another alteration detected in diabetic retina in our previous study affecting the synaptic terminals by an increase in synaptic proteins such as synaptophysin [27]. The upregulation of GFAP in Müller cells has been demonstrated in the retinas of *P*.*obesus* viewed as an indicator of retinal cell stress [27]. These changes, in combination with high blood glucose may unpair the function of these cells. Likewise, a high concentration of glucose can promote photoreceptor degeneration as demonstrated *in vitro* by Baccouche et al. [35]. ERG abnormalities found in the diurnal *P*.*obesus* were present for both scotopic (rod-mediated) and photopic (cone-mediated) conditions, indicating that both retinal systems are affected in our animal model of diabetic retinopathy. Based on ERG abnormalities and histologic findings in advanced human DR, a significant decrease in amplitudes of scotopic a- and b- waves from pre-proliferative DR [36] indicates impairment of the outer segments of the photoreceptors [37]. The dark-adapted ERG [38] is reported to be more impaired compared to the light-adapted ERG [39–41]. Thus, in rod dominated retinas, e.g. some rodents (97% rods in rats and mice) and peripheral human retina (96% rods), scotopic conditions that selectively measure rod responses allow easy detection of functional deficits [42]. Chung et al. [36] reported less temporal variability compared to amplitude variability of ERG and a significant prolonged b-wave implicit time at all stages of retinopathy and in the eyes of diabetic patients without retinopathy. Similar results have been observed in streptozotocin-induced diabetic rats [43–46] and in our model diabetics of *P*.*obesus* at 28 weeks after diabetes induction. A calculation of b-/a-wave amplitude ratios in the present study revealed a declined in scotopic condition, suggesting that bipolar cells are more affected than photoreceptors. However, in photopic condition the unchanged ratio showed that the decrease of b-wave amplitude was correlated to the photoreceptoral response.

### Impairment of individual OP wavelets

Our results also provide evidence of retinal impairments beyond the level of the photoreceptors in rodents with diabetes, given that OPs were shown to signal inner retinal activity, particularly the amacrine cells [47, 48]. Alteration of OPs in diabetic *P*.*obesus* may be explained by the loss of amacrine cells numbers at different cell retinal layers including retinal ganglion cells [27]. These changes were accompanied by an increase of immunohistochemical staining of tyrosine hydroxylase in amacrine cells of diabetes animals in comparison with control animals. As previously shown with clinical and experimental data [14, 49–51], the OPs are significantly altered in advanced stages of diabetes. The reduction in amplitude and delay in implicit time for each OP of the diabetic *P*.*obesus* ERG were comparable to what is reported in human patients at the proliferative stage in diabetic retinopathy [52–54]. This study confirms the previously reported link between attenuated and delayed OPs and severity of diabetic retinopathy in patients [19, 20, 55–57], a feature also observed in *P*.*obesus*.

Currently available diabetic rodent models can be used to study the initial (acute) or latent phase of diabetic retinopathy and several studies have reported a concurrent change in OPs [21, 58, 59]. For example, male Sprague-Dawley STZ rats showed an amplitude reduction of OP1 and OP2 after 6–20 weeks of diabetes induction [60]. However, contrasting with the above, spontaneous Torii rats showed a non-specific attenuation of all ERG components (i.e. a- and b-waves and the OPs) [15]. Our results thus show that our diet-induced diabetic type 2 *P*.*obesus* model mimics several of the DR features observed in human diabetes and should therefore be considered as a valid alternative to test therapeutic pharmacological molecules on type 2 DR functions.

### i-wave in diabetic retinopathy

The i-wave of the human full-field photopic ERG response is a relatively small positive deflection following the b-wave and is thought to originate in an off-circuitry in the inner retina [61]. Although Rosolen at al. [62] were able to record an i-wave in several species (dog, cat, rabbit, minipig, monkey) they were not able to detect it in mice and rats. However, the i-wave was clearly detectable in the Mongolian gerbil (a mostly diurnal rodent) by Yang et al. [63]. As the Mongolian gerbil retina has a higher proportion of cone photoreceptors (~13% [64]) vs. rats (0.85–1.5% [65], [66]), it is likely that the i-wave is generated by a more sophisticated cone circuitry compared to the one present in the standard laboratory rats. In support of this view, our results clearly demonstrate presence of an i-wave in the sand rat, which is also a mostly diurnal rodent and has a larger proportion of cone photoreceptors (41% [31]). This post b-wave component, which is said to originate from the activity of the retinal ganglion cells, probably the off pathway [67] became affected in diabetic *P*.*obesus* after 7 months of diabetic induction.

### Changes in PhNR

Degeneration of the retinal ganglion cells (RGC) and deterioration of the inner nuclear layer in the retinas of diabetic *P*.*obesus* were previously reported by our laboratory showing a reduction by 44% of RGCs in diabetic *P*.*obesus* retina after 7 months of diabetes progression [27]. In the present study, we show a~57% decrease in amplitude of the PhNR which is consistent with the hypothesis that reduced ganglion cells function may also be an important component of the pathogenesis of diabetic retinopathy, given that this negative, post b-wave component of the ERG is claimed to signal electrical events evoked in part the by RGC [68, 69]. In human patients, PhNR of reduced amplitude and increased timing were found to strongly correlate with progression of DR [14, 70, 71]. In the present study, we found that the PhNR was the ERG wave among the most severely affected component, suggesting that it could represent a more sensitive detector of DR onset.

### 30 HZ flicker alteration

Our results also showed that the photopic flicker ERG of our diabetic *P*.*obesus* was reduced in amplitude and delayed in timing, similar to what was previously reported in human diabetes [56, 72, 73]. Similar timing delays have been reported for other vascular retinopathy such as central and branch retinal vein occlusions [1]. The timing delays in the flash ERG and flicker can be explained by reduction in retinal sensitivity. In human Satoh et al. [74] described that the photopic ERG response of diabetic patients showed a clear alteration of the implicit time and a correlation of the amplitude with the severity of the disease. Based on the data presented in Table 2, a significant decrease has been reported in 30 Hz flicker. The reduction of flicker response seems to be the second more affected photopic component after OP 3. Based on data in Table 1, it also looks that the decrease in amplitude was more significant than delay in timing. Thus, it appears that photopic 30 Hz flicker amplitude flicker is a sensitive measure for the diet-induced DR progression in the *P*.*obesus*. Recognition of these defects will enhance our understanding of the pathophysiology of diabetic retinopathy.

### S-cone sensitivity loss

Finally, as previously shown in diabetic human subjects [75, 76], the S-cone ERGs of diabetic *P*.*obesus* was significantly reduced in amplitude and delayed in timing. The cone short-wavelength opsin staining was detectably reduced in *P*.*obesus* diabetic retinas and this was confirmed by Western blot analysis in our previous study [27].

In summary, the totally of our findings regarding ERG presented in this report further confirms the similarity between the characteristics of retina changes in diabetic *P*.*obesus* and the corresponding changes in human DR and support the notion that it represents a valid translational model to study the retinal pathophysiological processes involved in the onset and progression of type 2 diabetic retinopathy.”

Clearly, more studies are needed to better correlate structural and functional changes in the diabetic retina of *P*.*obesus*. Such studies should include fundus photography, optical coherence tomography and fluorescein angiography in order to clarify to what extent the ERG findings correlate (precede or follow) with morphological changes in the retina.

## Conclusion

The present study clearly demonstrated for the first time that long-lasting and significant alterations in visual function detected by full-field ERG take place after 28 weeks of diet-induced type 2 diabetes in the retina of the sand rat. Thus, the diabetic sand rat appears to be an animal model that mimics several important features of the human form of diabetic retinopathy.

Our results confirm the validity of the *P*.*obesus* as a useful translational model to study diet-induced type 2 diabetic retinopathy (induced, like in humans, with the increase intake of high caloric food). We strongly believe that adding this new model to the researchers’ armamentarium will not only be instrumental in increasing our understanding of the pathophysiology of human diabetic retinopathy but also help in the development of new therapeutic strategies.

## Supporting information

S1 Fig Individual presentation of retina function (electroretinograms) of *Psammomys obesus*, control 1, at 28 weeks following the onset of diabetes. Representative traces indicating: (A) Rod responses using 0.01 cd.s/m^2^ flash. (B) Mixed response using 3 cd.s/m^2^ flash. (C) Photopic responses using 3 cd.s/m^2^ flash. (D) Photopic 30 Hz flicker response. (E) Photopic S-cone response using 0.0045cd.s/m^2^ blue flash on an orange background.
(TIF)

S2 Fig Individual presentation of retina function (electroretinograms) of *Psammomys obesus*, control 2, at 28 weeks following the onset of diabetes. Representative traces indicating: (A) Rod responses using 0.01 cd.s/m^2^ flash. (B) Mixed response using 3 cd.s/m^2^ flash. (C) Photopic responses using 3 cd.s/m^2^ flash. (D) Photopic 30 Hz flicker response. (E) Photopic S-cone response using 0.0045cd.s/m^2^ blue flash on an orange background.
(TIF)

S3 Fig Individual presentation of retina function (electroretinograms) of *Psammomys obesus*, control 3, at 28 weeks following the onset of diabetes. Representative traces indicating: (A) Rod responses using 0.01 cd.s/m^2^ flash. (B) Mixed response using 3 cd.s/m^2^ flash. (C) Photopic responses using 3 cd.s/m^2^ flash. (D) Photopic 30 Hz flicker response. (E) Photopic S-cone response using 0.0045cd.s/m^2^ blue flash on an orange background.
(TIF)

S4 Fig Individual presentation of retina function (electroretinograms) of *Psammomys obesus*, control 4, at 28 weeks following the onset of diabetes. Representative traces indicating: (A) Rod responses using 0.01 cd.s/m^2^ flash. (B) Mixed response using 3 cd.s/m^2^ flash. (C) Photopic responses using 3 cd.s/m^2^ flash. (D) Photopic 30 Hz flicker response. (E) Photopic S-cone response using 0.0045cd.s/m^2^ blue flash on an orange background.
(TIF)

S5 Fig Individual presentation of retina function (electroretinograms) of *Psammomys obesus*, control 5, at 28 weeks following the onset of diabetes. Representative traces indicating: (A) Rod responses using 0.01 cd.s/m^2^ flash. (B) Mixed response using 3 cd.s/m^2^ flash. (C) Photopic responses using 3 cd.s/m^2^ flash. (D) Photopic 30 Hz flicker response. (E) Photopic S-cone response using 0.0045cd.s/m^2^ blue flash on an orange background.
(TIF)

S6 Fig Individual presentation of retina function (electroretinograms) of *Psammomys obesus*, control 6, at 28 weeks following the onset of diabetes. Representative traces indicating: (A) Rod responses using 0.01 cd.s/m^2^ flash. (B) Mixed response using 3 cd.s/m^2^ flash. (C) Photopic responses using 3 cd.s/m^2^ flash. (D) Photopic 30 Hz flicker response. (E) Photopic S-cone response using 0.0045cd.s/m^2^ blue flash on an orange background.
(TIF)

S7 Fig Individual presentation of retina function (electroretinograms) of *Psammomys obesus*, diabetic 1, at 28 weeks following the onset of diabetes. Representative traces indicating: (A) Rod responses using 0.01 cd.s/m^2^ flash. (B) Mixed response using 3 cd.s/m^2^ flash. (C) Photopic responses using 3 cd.s/m^2^ flash. (D) Photopic 30 Hz flicker response. (E) Photopic S-cone response using 0.0045cd.s/m^2^ blue flash on an orange background.
(TIF)

S8 Fig Individual presentation of retina function (electroretinograms) of *Psammomys obesus*, diabetic 2, at 28 weeks following the onset of diabetes. Representative traces indicating: (A) Rod responses using 0.01 cd.s/m^2^ flash. (B) Mixed response using 3 cd.s/m^2^ flash. (C) Photopic responses using 3 cd.s/m^2^ flash. (D) Photopic 30 Hz flicker response. (E) Photopic S-cone response using 0.0045cd.s/m^2^ blue flash on an orange background.
(TIF)

S9 Fig Individual presentation of retina function (electroretinograms) of *Psammomys obesus*, diabetic 3, at 28 weeks following the onset of diabetes. Representative traces indicating: (A) Rod responses using 0.01 cd.s/m^2^ flash. (B) Mixed response using 3 cd.s/m^2^ flash. (C) Photopic responses using 3 cd.s/m^2^ flash. (D) Photopic 30 Hz flicker response. (E) Photopic S-cone response using 0.0045cd.s/m^2^ blue flash on an orange background.
(TIF)

S10 Fig Individual presentation of retina function (electroretinograms) of *Psammomys obesus*, diabetic 4, at 28 weeks following the onset of diabetes. Representative traces indicating: (A) Rod responses using 0.01 cd.s/m^2^ flash. (B) Mixed response using 3 cd.s/m^2^ flash. (C) Photopic responses using 3 cd.s/m^2^ flash. (D) Photopic 30 Hz flicker response. (E) Photopic S-cone response using 0.0045cd.s/m^2^ blue flash on an orange background.
(TIF)

S11 Fig Individual presentation of retina function (electroretinograms) of *Psammomys obesus*, diabetic 5, at 28 weeks following the onset of diabetes. Representative traces indicating: (A) Rod responses using 0.01 cd.s/m^2^ flash. (B) Mixed response using 3 cd.s/m^2^ flash. (C) Photopic responses using 3 cd.s/m^2^ flash. (D) Photopic 30 Hz flicker response. (E) Photopic S-cone response using 0.0045cd.s/m^2^ blue flash on an orange background.
(TIF)

S12 Fig Individual presentation of retina function (electroretinograms) of *Psammomys obesus*, diabetic 6, at 28 weeks following the onset of diabetes. Representative traces indicating: (A) Rod responses using 0.01 cd.s/m^2^ flash. (B) Mixed response using 3 cd.s/m^2^ flash. (C) Photopic responses using 3 cd.s/m^2^ flash. (D) Photopic 30 Hz flicker response. (E) Photopic S-cone response using 0.0045cd.s/m^2^ blue flash on an orange background.
(TIF)
