# Supplementary figures and images for: Electroretinographic evidence suggesting that the type 2 diabetic retinopathy of the sand rat Psammomys obesus is comparable to that of humans
Source: PLoS One. 2018 Feb 8;13(2):e0192400. doi: 10.1371/journal.pone.0192400 (PMC5805270; doi:10.1371/journal.pone.0192400)

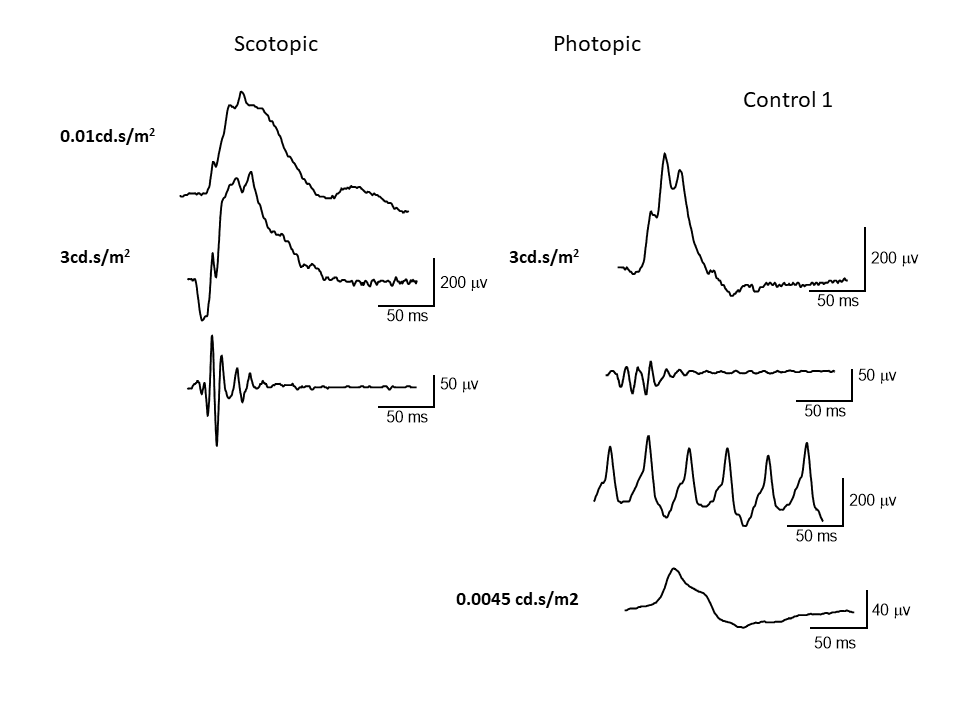

Supplement: S1 Fig — Representative traces indicating: (A) Rod responses using 0.01 cd.s/m2 flash. (B) Mixed response using 3 cd.s/m2 flash. (C) Photopic responses using 3 cd.s/m2 flash. (D) Photopic 30 Hz flicker response. (E) Photopic S-cone response using 0.0045cd.s/m2 blue flash on an orange background. (TIF) [file pone.0192400.s001.tif]

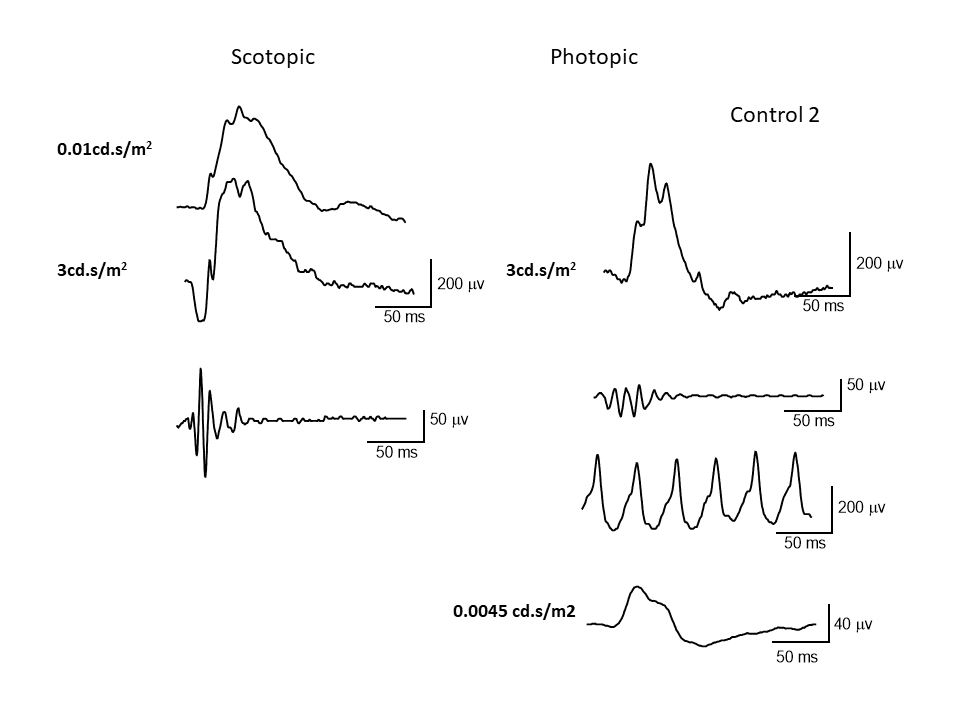

Supplement: S2 Fig — Representative traces indicating: (A) Rod responses using 0.01 cd.s/m2 flash. (B) Mixed response using 3 cd.s/m2 flash. (C) Photopic responses using 3 cd.s/m2 flash. (D) Photopic 30 Hz flicker response. (E) Photopic S-cone response using 0.0045cd.s/m2 blue flash on an orange background. (TIF) [file pone.0192400.s002.tif]

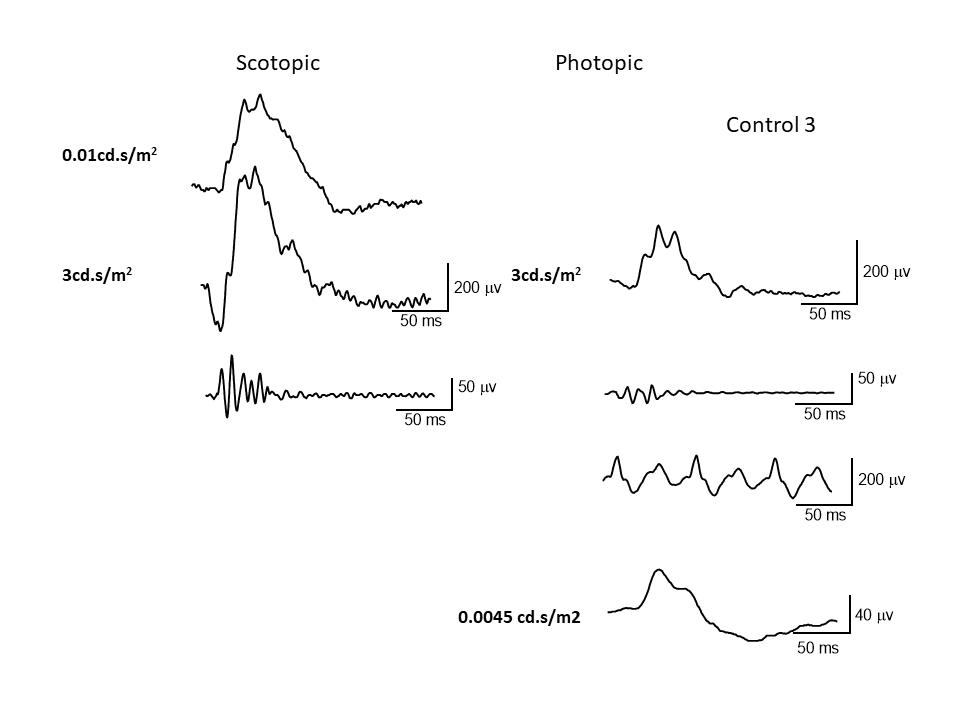

Supplement: S3 Fig — Representative traces indicating: (A) Rod responses using 0.01 cd.s/m2 flash. (B) Mixed response using 3 cd.s/m2 flash. (C) Photopic responses using 3 cd.s/m2 flash. (D) Photopic 30 Hz flicker response. (E) Photopic S-cone response using 0.0045cd.s/m2 blue flash on an orange background. (TIF) [file pone.0192400.s003.tif]

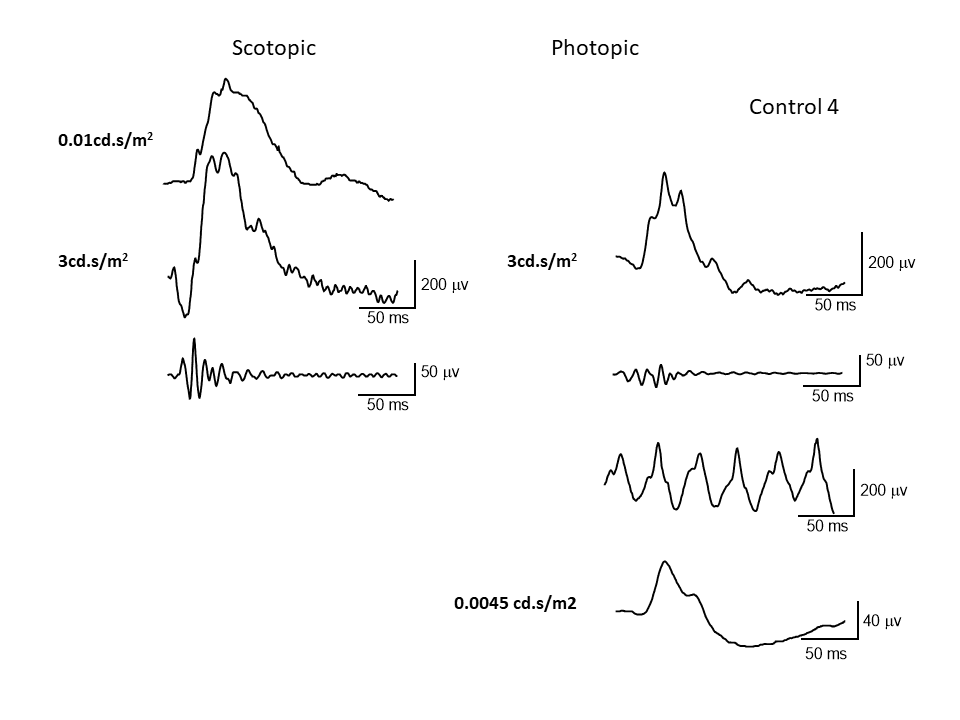

Supplement: S4 Fig — Representative traces indicating: (A) Rod responses using 0.01 cd.s/m2 flash. (B) Mixed response using 3 cd.s/m2 flash. (C) Photopic responses using 3 cd.s/m2 flash. (D) Photopic 30 Hz flicker response. (E) Photopic S-cone response using 0.0045cd.s/m2 blue flash on an orange background. (TIF) [file pone.0192400.s004.tif]

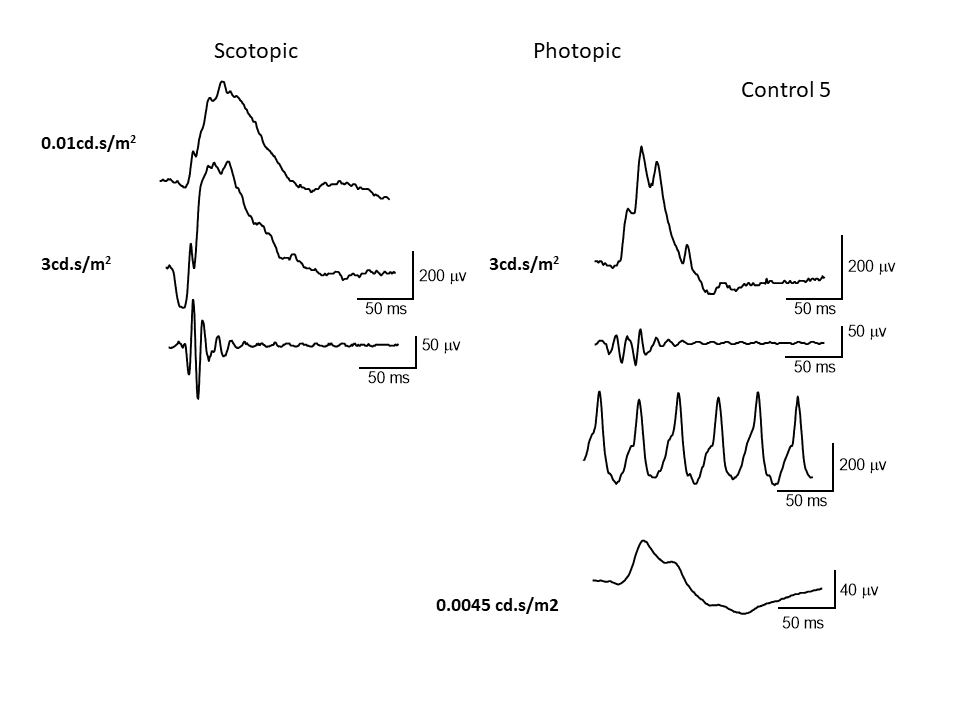

Supplement: S5 Fig — Representative traces indicating: (A) Rod responses using 0.01 cd.s/m2 flash. (B) Mixed response using 3 cd.s/m2 flash. (C) Photopic responses using 3 cd.s/m2 flash. (D) Photopic 30 Hz flicker response. (E) Photopic S-cone response using 0.0045cd.s/m2 blue flash on an orange background. (TIF) [file pone.0192400.s005.tif]

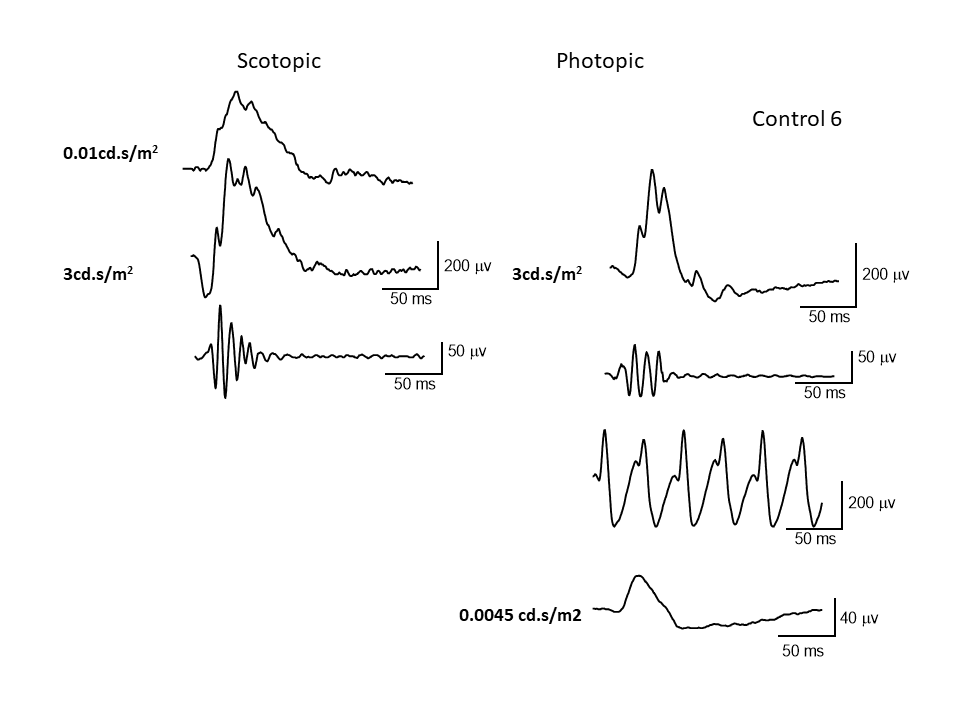

Supplement: S6 Fig — Representative traces indicating: (A) Rod responses using 0.01 cd.s/m2 flash. (B) Mixed response using 3 cd.s/m2 flash. (C) Photopic responses using 3 cd.s/m2 flash. (D) Photopic 30 Hz flicker response. (E) Photopic S-cone response using 0.0045cd.s/m2 blue flash on an orange background. (TIF) [file pone.0192400.s006.tif]

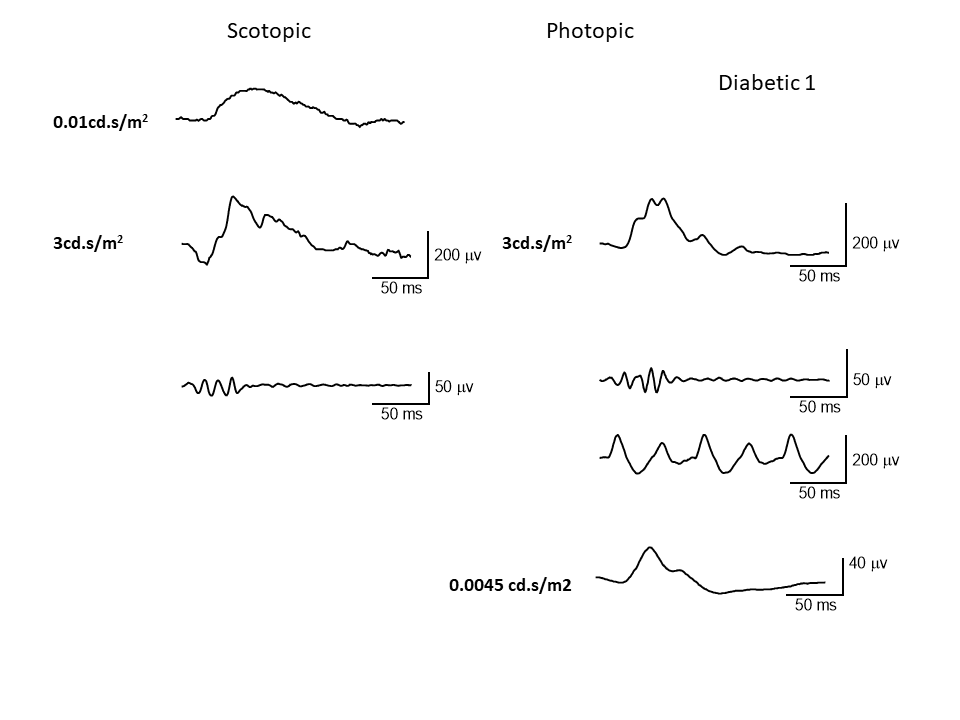

Supplement: S7 Fig — Representative traces indicating: (A) Rod responses using 0.01 cd.s/m2 flash. (B) Mixed response using 3 cd.s/m2 flash. (C) Photopic responses using 3 cd.s/m2 flash. (D) Photopic 30 Hz flicker response. (E) Photopic S-cone response using 0.0045cd.s/m2 blue flash on an orange background. (TIF) [file pone.0192400.s007.TIF]

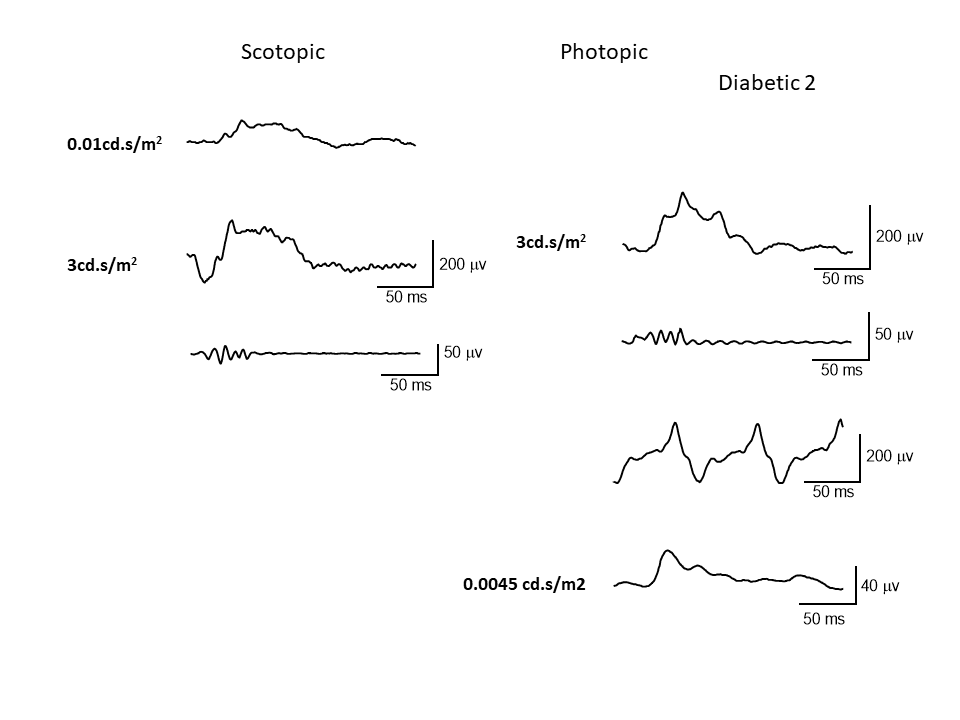

Supplement: S8 Fig — Representative traces indicating: (A) Rod responses using 0.01 cd.s/m2 flash. (B) Mixed response using 3 cd.s/m2 flash. (C) Photopic responses using 3 cd.s/m2 flash. (D) Photopic 30 Hz flicker response. (E) Photopic S-cone response using 0.0045cd.s/m2 blue flash on an orange background. (TIF) [file pone.0192400.s008.TIF]

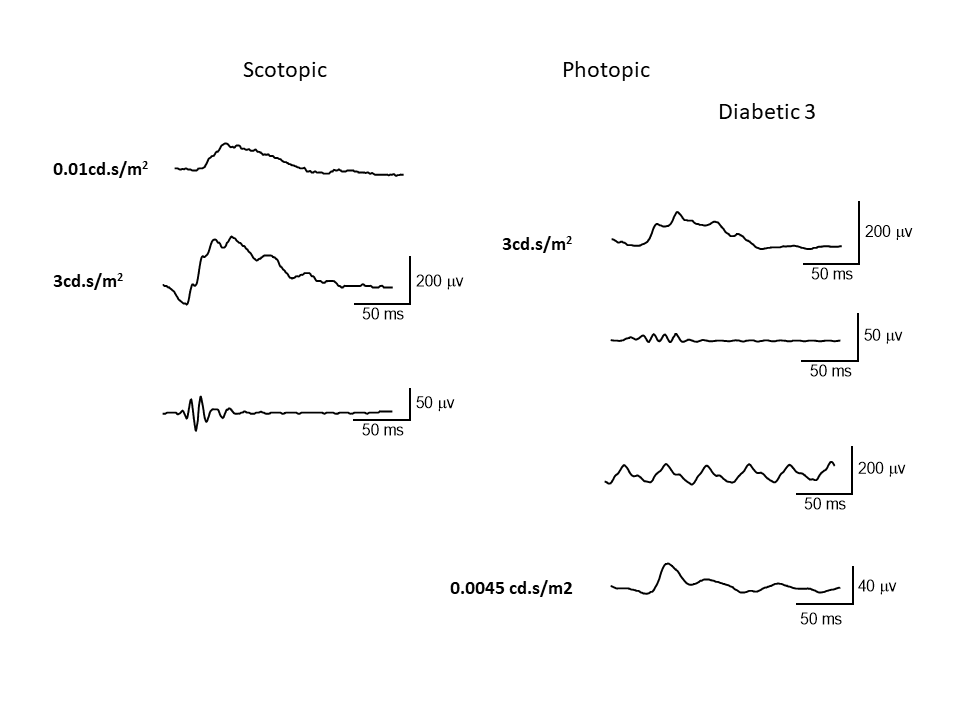

Supplement: S9 Fig — Representative traces indicating: (A) Rod responses using 0.01 cd.s/m2 flash. (B) Mixed response using 3 cd.s/m2 flash. (C) Photopic responses using 3 cd.s/m2 flash. (D) Photopic 30 Hz flicker response. (E) Photopic S-cone response using 0.0045cd.s/m2 blue flash on an orange background. (TIF) [file pone.0192400.s009.TIF]

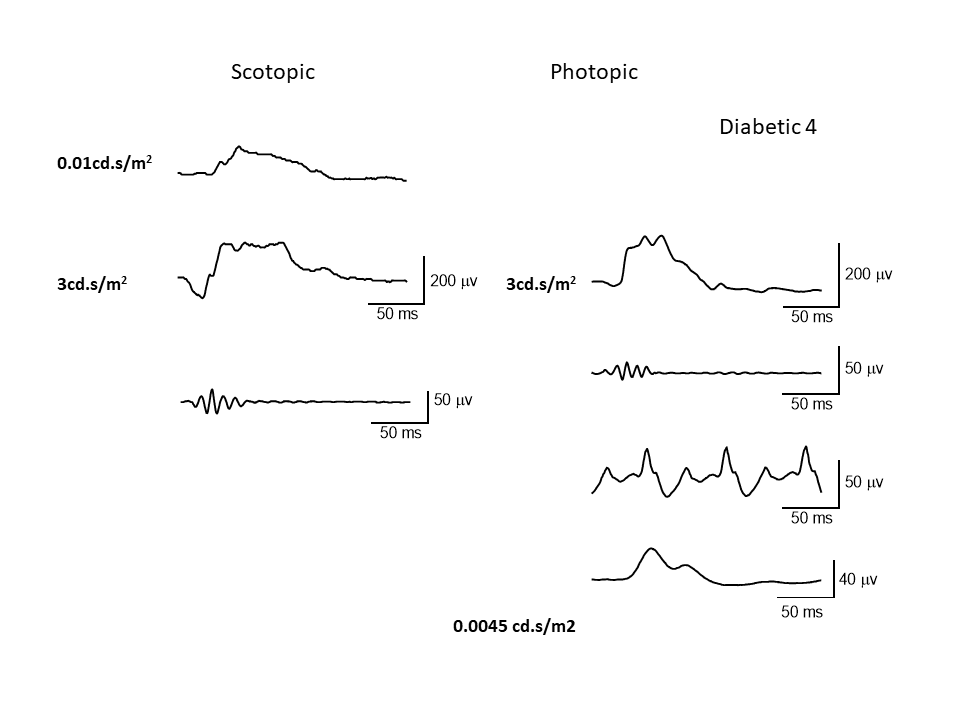

Supplement: S10 Fig — Representative traces indicating: (A) Rod responses using 0.01 cd.s/m2 flash. (B) Mixed response using 3 cd.s/m2 flash. (C) Photopic responses using 3 cd.s/m2 flash. (D) Photopic 30 Hz flicker response. (E) Photopic S-cone response using 0.0045cd.s/m2 blue flash on an orange background. (TIF) [file pone.0192400.s010.TIF]

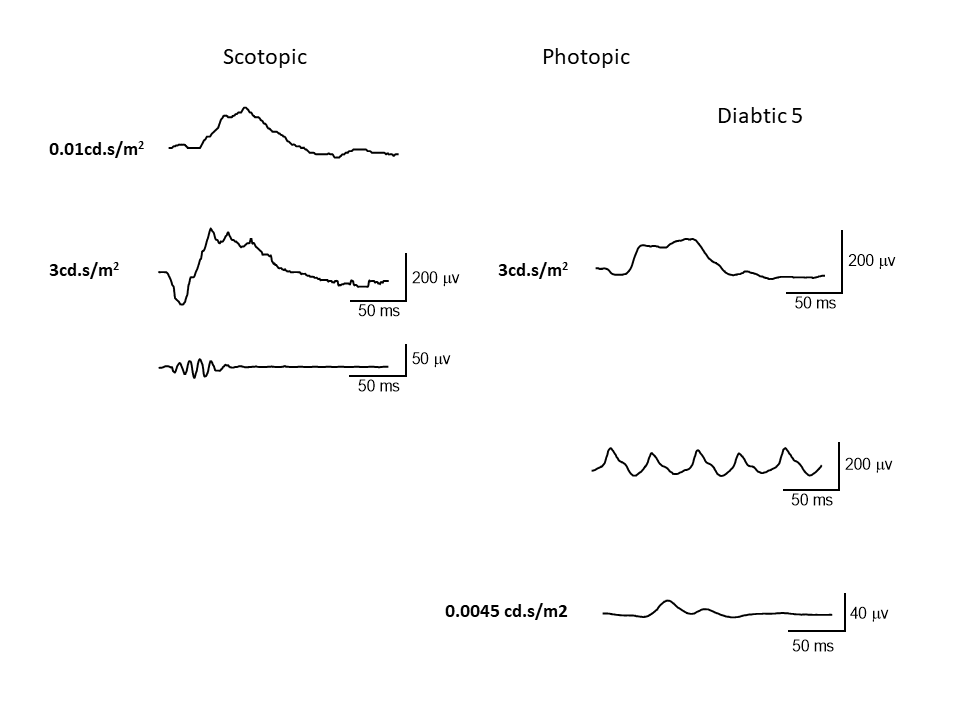

Supplement: S11 Fig — Representative traces indicating: (A) Rod responses using 0.01 cd.s/m2 flash. (B) Mixed response using 3 cd.s/m2 flash. (C) Photopic responses using 3 cd.s/m2 flash. (D) Photopic 30 Hz flicker response. (E) Photopic S-cone response using 0.0045cd.s/m2 blue flash on an orange background. (TIF) [file pone.0192400.s011.TIF]

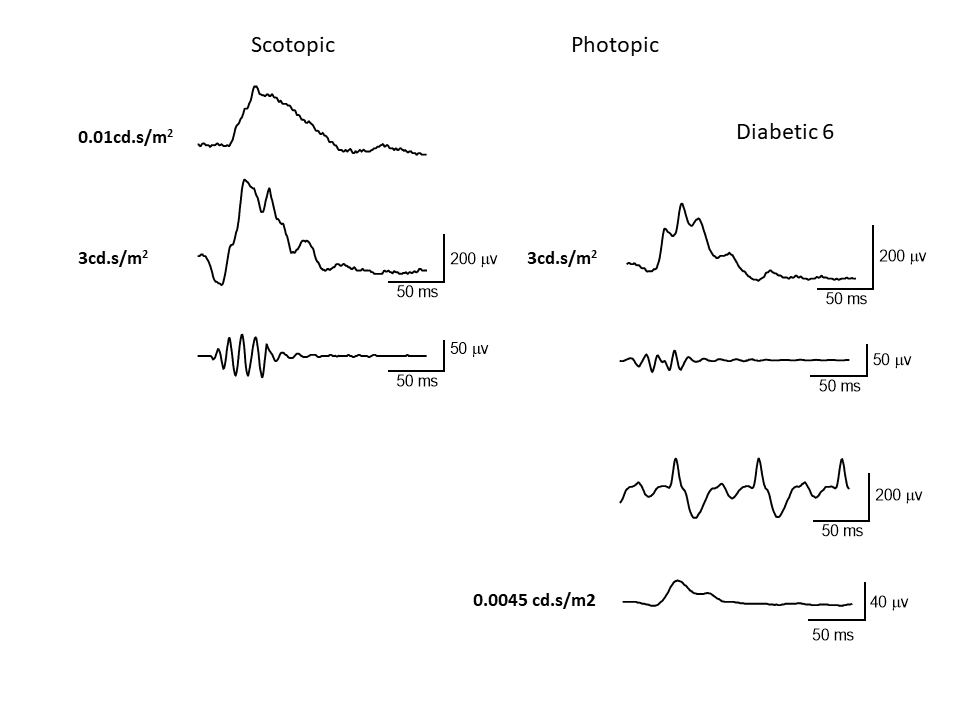

Supplement: S12 Fig — Representative traces indicating: (A) Rod responses using 0.01 cd.s/m2 flash. (B) Mixed response using 3 cd.s/m2 flash. (C) Photopic responses using 3 cd.s/m2 flash. (D) Photopic 30 Hz flicker response. (E) Photopic S-cone response using 0.0045cd.s/m2 blue flash on an orange background. (TIF) [file pone.0192400.s012.TIF]
